# Towards a consensus on genotyping schemes for surveillance and outbreak investigations of *Cryptosporidium*, Berlin, June 2016

**DOI:** 10.2807/1560-7917.ES.2016.21.37.30338

**Published:** 2016-09-15

**Authors:** RM Chalmers, S Cacciò

**Affiliations:** 1 Cryptosporidium Reference Unit, Public Health Wales Microbiology and Health Protection, Singleton Hospital, Swansea, United Kingdom; 2 Swansea University Medical School, Singleton Park, Swansea, Wales, United Kingdom; 3 European Union reference laboratory for Parasites, Istituto Superiore di Sanità, Rome, Italy

**Keywords:** cryptosporidiosis, multilocus, standardised, outbreaks, molecular epidemiology

This report outlines the evidence and main conclusions presented at an expert workshop on *Cryptosporidium* genotyping held on 16 and 17 June 2016, hosted by the Robert Koch Institute, Berlin, and funded by EU COST Action FA1408 “A European Network for Foodborne Parasites: Euro-FBP” (http://www.euro-fbp.org).

The consultation brought together 23 scientists and experts in public and animal health from 12 European countries and the United States (US) to discuss how *Cryptosporidium* spp. surveillance and outbreak investigations could benefit from a harmonised approach to intra-species differentiation of the two main human pathogens, *C. parvum* and *C. hominis.* These are major zoonotic and anthroponotic causes of gastroenteritis, respectively. There is currently no standardised genotyping scheme for these protozoan parasites.

The workshop was organised in two parts: firstly, specialists described the current state of knowledge and need, and secondly, four working groups considered different aspects of the development, implementation and maintenance of *Cryptosporidium* genotyping schemes.

## An overview of genotyping *Cryptosporidium* for public health purposes

Laetitia Kortbeek (National Institute for Public Health and the Environment, the Netherlands) described the diagnosis of *Cryptosporidium* and the usefulness of genotyping for epidemiology. Although cryptosporidiosis cases are notifiable in some European Union (EU) countries, testing and diagnostic practices are variable. Improved understanding of the epidemiology, sources and transmission of cryptosporidiosis is needed, but surveillance is also highly variable and the quality of the data provided to the European Centre for Disease Prevention and Control (ECDC) hinders comparisons between countries [1]. Improved diagnosis and basic surveillance across the EU would provide the means to estimate and compare the prevalence of cryptosporidiosis and detect changing trends in transmission.

The complexity of *Cryptosporidium* transmission was highlighted using data from the Netherlands, where a proportion of *Cryptosporidium-*positive stools are genotyped to identify species. In the second half of 2012, an excess of cases, mainly due to *C. hominis,* triggered an alert to other EU countries via ECDC’s Epidemic Intelligence Information System for Food and Waterborne Diseases (EPIS); the United Kingdom (UK) and Germany also reported an increase [2]. An ongoing case–control study in the Netherlands failed to reveal an endemic source. In the following year, *C. parvum* predominated and risk factors for infection included the use of inland bathing waters and animal contact (not unexpected for *C. parvum*). More discriminatory genotyping of isolates could contribute to the identification of parasite sources and routes of transmission. As a first step, partial sequencing of a gene encoding a highly variable surface antigen (gp60) has shown that *C. hominis* allele IbA10G2 is highly prevalent throughout Europe, whereas *C. parvum* has greater diversity at this locus [3]. There is no specific licensed treatment in the EU for cryptosporidiosis, so understanding the epidemiology and improving the ability to identify sources through genotyping are important for the interruption of transmission routes and subsequent disease reduction.

## The confusing world of *Cryptosporidium* typing

Giovanni Widmer (Tufts University, US) described how consideration of the reproductive biology and genetics of the parasite and analysis of metadata from studies that used the same genotyping markers have provided further clarification of *Cryptosporidium* diversity, especially within *C. parvum*. The lifecycle involves asexual and sexual reproductive stages, requiring a multilocus scheme to account for sexual recombination within genetically diverse populations. Therefore, it is important to select markers that are sufficiently distant or located on different chromosomes, to ensure they are not in linkage. Excluding markers that provide redundant information reduces wastage and increases efficiency. As part of the marker selection process, ordination methods such as principal coordinates analysis and rank abundance plots can be used to estimate objectively how informative individual genetic markers and their combinations are. Because of the multivariate nature of multilocus data, ordination methods are ideal to visualise genetic similarity among isolates [4] and infer the likely source of an outbreak. In silico analysis of existing data can be used to improve and harmonise current genotyping approaches for surveillance and outbreak investigations.

## Human epidemiology and food-borne outbreaks

Rachel Chalmers (National Cryptosporidium Reference Unit, UK) showed how supplementing epidemiological and environmental data with *Cryptosporidium* species and gp60 allele identification has strengthened the statistical evidence of association with food exposures in outbreaks. In May 2012, an excess of 300 cases of *C. parvum* was linked to the consumption of pre-cut mixed salad leaves, spinach and tomatoes [5]. The odds of association with eating pre-cut mixed salad leaves were increased when the case definition was restricted to those infected with gp60 allele IIaA15G2R1. In 2015, *C. hominis* infections exceeded expected numbers by more than 900 cases in late summer/early autumn, triggering an EPIS alert, with a similar increase reported by the Netherlands. Hypothesis-generating questionnaires revealed no sufficiently common exposures or risk factors to allow a case–control study. Isolates with the gp60 allele IbA10G2 predominated. Not only is this allele highly prevalent among *C. hominis* isolates from northern Europe, but there is also limited heterogeneity at other loci, highlighting the limitation of multilocus genotyping as an epidemiological tool for this species [3]. Suitable samples [6] with the IbA10G2 allele were further analysed by whole genome sequencing. Very few differences were seen in pairwise comparisons, with at most 50 single nucleotide polymorphisms (SNPs) observed in the ca 9.2 Mbp genome; the significance of these extremely small differences is currently unknown. In contrast, a *C. parvum* outbreak of more than 300 cases at the end of 2015 was defined by an unusual gp60 allele, IIdA24G1, recognised initially by the Scottish Parasite Diagnostic and Reference Laboratory, highlighting the value of genotyping routinely and including the data in national surveillance. A case–control study revealed food-linked exposures and the outbreak remains under investigation at the time of writing, demonstrating the difficulties in food chain investigations.

## Zoonotic transmission

Karin Troell (National Veterinary Institute, Sweden) illustrated the importance of applying One Health approaches to the investigation of *Cryptosporidium* as a zoonosis. In Sweden, samples are tested from any likely host animal that is linked to a human cryptosporidiosis case, for example from household cats when *C. felis* has been detected in a patient [7]. This has led to collaborative studies on other, less common, species causing human infections. These findings reinforce the need for clinical diagnostics to detect not only *C. parvum* and *C. hominis*.

The most common zoonotic species in humans, *C. parvum*, has an unusual epidemiology in cattle in Sweden, where some studies have shown low prevalence even in young calves. This is in contrast to other countries where *C. parvum* is the main cause of cryptosporidiosis in pre-weaned calves [8]. Despite this, one of the most common *C. parvum *gp60 alleles in cattle, IIaA16G1R1, is also frequent in humans in Sweden. To support epidemiological investigations, a multilocus sequencing tool based on nine SNP markers across five chromosomes has been evaluated in a multiplex PCR on numerous samples; high discriminatory power and evidence of transmission between calves and humans in Sweden was shown.**However, further studies of the population structure of *C. parvum* are needed across Europe to assess the broader applicability of this scheme.

## How diversity relates to transmission to humans

Simone Cacciò (National Institute of Health, Italy) described the apparent geographic diversity of *C. parvum* in Ireland, Italy, and Scotland, as revealed by multilocus analyses. Studies so far indicate that in those countries, *C. parvum* populations from humans and livestock may have become isolated from each other, to the extent that the opportunity for genetic interchange appears limited [9]. To investigate the degree of genetic isolation, further studies are needed across Europe that include the major hosts for *C. parvum*. One study showed that in the UK, a high proportion of *C. hominis *isolates were indistinguishable at multiple loci, contrasting with those from Uganda, where a more diverse population structure was found [10]. Therefore, conclusions from one location may not be widely applicable and information is specific to host populations, whether these are defined geographically or demographically. A European-wide project (COMPARE; http://www.compare-europe.eu/) aims to increase the number of whole genome sequences for *Cryptosporidium* and to develop bioinformatic pipelines that would further the understanding of the population biology and determinants of virulence of the parasite. Information from COMPARE will undoubtedly benefit typing scheme development.

Four working groups considered how the evidence presented could be used to develop, implement and maintain suitable genotyping resources for *Cryptosporidium*.

## Are the genetic and population structures of *Cryptosporidium* amenable to developing a genotyping scheme?

One working group considered whether reliable predictions of transmission can be made by combining genotyping with epidemiological and clinical data, considering that genetic diversity and population structures differ for *C. parvum* and *C. hominis*. It concluded that data are currently unavailable for much of Europe and are often not comparable because of lack of standardisation, indicating the need for further studies. Sampling frames need to follow the One Health concept, including both human and animal samples. Comparative analysis of increasingly available genome sequence data can provide a solid basis for marker selection. An evaluation process should be defined and applied to those markers already used.

## What needs to be done to develop a standardised, multilocus genotyping scheme?

Another working group considered the development of separate multilocus schemes for *C. parvum* and *C. hominis* to provide robust, cost-effective assays, suitable for specialist and reference laboratories. Fragment sizing of regions containing tandem nucleotide repeats was considered alongside in-house sequencing. The decision whether to choose fragment sizing or sequencing will depend on the best workflow for individual laboratories, but markers that provide the same results with either method would be desirable. Sequence data from gp60 remains important. The most suitable markers need to be identified through a structured and objective process, ideally starting from whole-genome comparisons. Well-defined panels of samples are needed for biological and statistical evaluation of individual markers and their combinations, before progressing to inter-laboratory trials. DNA standards should be available. A web-based database needs be developed to contextualise metadata and genetic identification of isolates.

## A multilocus genotyping scheme as a component of epidemic preparedness and response

A third working group considered multilocus genotyping as a component of a resilient response for health protection, highlighting that any scheme should be informative for epidemiological investigations and the detection and management of outbreaks, and that genotyping results should be incorporated into the collection of high quality epidemiological data. Differentiating between what is ‘nice to know’ and ‘essential to know’ is important: at present, there is more to be gained from genotyping *C. parvum,* as a high proportion of *C. hominis* cases in Europe have the gp60 allele IbA10G2, which is associated with low diversity at other markers. If genotyping all cases cannot be justified, selection will depend on outbreak size and available information and is probably best delivered as a test done in specialist or reference laboratories. Simulated outbreak exercises should be undertaken.

## Sustainability of a standardised, multilocus genotyping scheme

The final working group discussed the elements needed to sustain a standardised scheme, including validation, external quality control (EQA), and inclusion of future developments, for example identification of new informative markers. A good mechanism for EQA should be established using an independent provider, also providing training modules and DNA standards. Central, ongoing collection of a minimum set of metadata are needed to facilitate surveillance of genotypes and meaningful comparisons and interpretation; this may be possible through the *Cryptosporidium* database at http://CryptoDB.org. Nomenclature for multilocus genotypes needs to be adopted for effective interdisciplinary communication.

## Conclusions

Increased standardisation of diagnostic practices for *Cryptosporidium* is fundamental to the meaningful interpretation of surveillance data and distribution of species and genotypes. A robust, standardised, multilocus genotyping scheme should be developed, using a defined process to replace or supplement the multitude of genotyping methods used. Although further genotyping of *C. parvum* would be highly informative, this procedure may not always be warranted for the genetically more conserved *C. hominis* in Europe. A web-based database, enabling interpretation of genotype occurrence and distribution trends in an epidemiological context, is required. Genotype data should be incorporated into national surveillance programmes, and a standardised nomenclature provided for effective communication with public health professionals.

## References

[1] European Centre for Disease Prevention and Control (ECDC). Annual epidemiological report 2014 –food- and waterborne diseases and zoonoses. Stockholm: ECDC; 2014. Available from: http://ecdc.europa.eu/en/publications/Publications/food-waterborne-diseases-annual-epidemiological-report-2014.pdf

[2] Fournet N Deege MP Urbanus AT Nichols G Rosner BM Chalmers RM Simultaneous increase of Cryptosporidium infections in the Netherlands, the United Kingdom and Germany in late summer season, 2012. Euro Surveill. 2013;18(2):20348. 23324424

[3] Cacciò SM Chalmers RM. Human cryptosporidiosis in Europe. Clin Microbiol Infect. 2016;22(6):471-80. 10.1016/j.cmi.2016.04.021 27172805

[4] Widmer G Lee Y. Comparison of single- and multilocus genetic diversity in the protozoan parasites Cryptosporidium parvum and C. hominis. Appl Environ Microbiol. 2010;76(19):6639-44. 10.1128/AEM.01268-10 20709840 PMC2950454

[5] McKerr C Adak GK Nichols G Gorton R Chalmers RM Kafatos G An outbreak of *Cryptosporidium parvum* across England & Scotland associated with consumption of fresh pre-cut salad leaves, May 2012. PLoS One. 2015;10(5):e0125955. 10.1371/journal.pone.0125955 26017538 PMC4446264

[6] Hadfield SJ Pachebat JA Swain MT Robinson G Cameron SJS Alexander J Generation of whole genome sequences of new Cryptosporidium hominis and Cryptosporidium parvum isolates directly from stool samples. BMC Genomics. 2015;16(1):650. 10.1186/s12864-015-1805-9 26318339 PMC4552982

[7] Beser J Toresson L Eitrem R Troell K Winiecka-Krusnell J Lebbad M. Possible zoonotic transmission of Cryptosporidium felis in a household. Infect Ecol Epidemiol. 2015;5(0):28463. 10.3402/iee.v5.28463 26446304 PMC4596888

[8] Björkman C Lindström L Oweson C Ahola H Troell K Axén C. Cryptosporidium infections in suckler herd beef calves. Parasitology. 2015;142(8):1108-14. 10.1017/S0031182015000426 25899555 PMC4453919

[9] Cacciò SM de Waele V Widmer G. Geographical segregation of Cryptosporidium parvum multilocus genotypes in Europe. Infect Genet Evol. 2015;31:245-9. 10.1016/j.meegid.2015.02.008 25687913

[10] Tanriverdi S Grinberg A Chalmers RM Hunter PR Petrovic Z Akiyoshi DE Inferences on the global population structure of Cryptosporidium parvum and Cryptosporidium hominis. Appl Environ Microbiol. 2008;74(23):7227-34. 10.1128/AEM.01576-08 18836013 PMC2592928

